# Pontiac fever: an operational definition for epidemiological studies

**DOI:** 10.1186/1471-2458-6-112

**Published:** 2006-04-28

**Authors:** Paul Tossa, Magali Deloge-Abarkan, Denis Zmirou-Navier, Philippe Hartemann, Laurence Mathieu

**Affiliations:** 1 Département Environnement et Santé Publique and INSERM ERI 11, Henri Poincaré University; Nancy, School of Medicine – 9 avenue de la Forêt de Haye, BP 184 – 54 505 Vandoeuvre lès Nancy, France; 2 Laboratoire d'Hydroclimatologie Médicale Santé Environnement, Ecole Pratique des Hautes Etudes (EPHE) and INSERM ERI 11; Nancy, School of Medicine – 9 avenue de la Forêt de Haye, BP 184 – 54 505 Vandoeuvre-lès-Nancy, France

## Abstract

**Background:**

Pontiac fever is usually described in epidemic settings. Detection of Pontiac fever is a marker of an environmental contamination by *Legionella *and should thereby call for prevention measures in order to prevent outbreak of Legionnaire's disease. The objective of this study is to propose an operational definition of Pontiac fever that is amenable to epidemiological surveillance and investigation in a non epidemic setting.

**Methods:**

A population of 560 elderly subjects residing in 25 nursing homes was followed during 4 months in order to assess the daily incidence of symptoms associated, in the literature, with Pontiac fever. The water and aerosol of one to 8 showers by nursing home were characterized combining conventional bacterial culture of *Legionella *and the Fluorescence In Situ Hybridization (FISH) technique that used oligonucleotides probes specific for *Legionellaceae*. A definition of Pontiac fever was devised based on clinical symptoms described in epidemic investigations and on their timing after the exposure event. The association between incidence of Pontiac fever and shower contamination levels was evaluated to test the relevance of this definition.

**Results:**

The proposed definition of Pontiac fever associated the following criteria: occurrence of at least one symptom among headache, myalgia, fever and shivers, possibly associated with other 'minor' symptoms, within three days after a shower contaminated by *Legionella*, during a maximum of 8 days (minimum 2 days). 23 such cases occurred during the study (incidence rate: 0.125 cases per person-year [95% CI: 0.122–0.127]). A concentration of *Legionella *in water equal to or greater than 10^4^.L^-1 ^(FISH method) was associated with a significant increase of incidence of Pontiac fever (p = 0.04).

**Conclusion:**

Once validated in other settings, the proposed definition of Pontiac fever might be used to develop epidemiological surveillance and help draw attention on sources of *Legionella*.

## Background

Pontiac fever (PF) is the mild form that takes infection by *Legionella*. It usually appears on an epidemic mode and is not associated with pneumonia [1]. Like for Legionaires' disease, infection stems from inhalation of an aerosol contaminated by *Legionella *[2]. Clinically, Pontiac fever's symptoms mimic influenza, with fever, asthenia, myalgia, arthralgia, headache, cough, nausea and sore throat [1,3]; other symptoms such as dyspnea, thoracic pains [2-4] vomiting and diarrhoea [3,5,6] have also been described. Patients recover in two to five days, without treatment [7-9].

Because of its benignity and lack of specificity, the occurrence of PF is often undiagnosed and is therefore less reported than Legionnaires' disease. Epidemiologically, PF is characterized by a short incubation period (typically 30 to 90 hours, with an average of 36 h), a high attack rate (70 to 90%) [1], and absence of fatalities or long term complications [2]. Age, gender and smoking do not seem to be risk factors [10,11]. Rather, PF seems to affect preferentially young subjects: the age of cases was 36 to 39 years in the original Pontiac episode [12,13], and age medians during different documented epidemics were 29 [4,10], 30 [3] and 32 years [11]. Pathogenesis of the PF is poorly known. To date, there is no consensus on the duration of the incubation period, on its clinical symptoms, nor on the causal species of *Legionella*.

Different serogroups (SGs) of *Legionella pneumophila *(*Lp*) (1, 6 and 7) [14-16], as well as *L. feeleii *[12,17], *L. micdadei *[2,11,12], *L. anisa *[13] can cause PF. In terms of diagnosis, according to some authors, PF develops the same serological characteristics as Legionnaires'disease [10,18]. Others claim on the contrary that serology during of a PF is inconstantly positive [19]. Presence of urinary antigen is not systematic either, even for epidemics connected to *Lp *SG 1 [10,20].

Detection of PF is a marker of an environmental contamination by *Legionella *and should thereby call for prevention measures. Efforts to standardize the definition of PF may facilitate comparison of risk levels and help draw attention on sources of *Legionella*. In this article, based on data from the Legion'Air project, we propose an operational definition of PF for the purpose of surveillance and epidemiological studies.

## Methods

The objectives of the Legion'Air project are: 1) to assess the exposure of elderly people residing in nursing homes to *Legionella *through aerosols generated by hot-water during showers, and 2) to evaluate the risk that is associated with this exposure.

Nursing homes solicited to participate in the Legion'Air project were located in the Lorraine region, north east of France. The selection process was based on the capacity of the nursing homes and on practical considerations (it should be located not too far from the study centre); no consideration was given to prior knowledge of contamination of the hot water system, in order to prevent selection bias.

This epidemiological study is a retrospective follow-up study. A population of 560 elderly volunteers (informed consent was obtained from patients or guardians) have been followed during 4 months. A set of predefined symptoms were registered daily by the auxiliary nursing staff, symptoms that had been previously reported in the literature in case of PF (table 1). Data were collected on demographic characteristics, current and/or past smoking habits, relevant medical history (respiratory and immunity-related conditions, such as diabetes and cancer) and current prescription of immunosuppressive therapy and of antibiotic medicines. A dedicated nurse insured the quality of recordings in the registers by alternating on site visits and telephone calls during the 4 months.

Volunteers included in the study were those who took at least one shower weekly. As shower practice represented the key exposure determinant (none of the participating nursing homes had air conditioning), data about shower habits were also recorded (the day and the room where the shower was taken).

At the end of the 4 months study, blood and urine samples were taken to assay anti *Legionella *antibodies and urine antigens of *Lp *SG 1. The antibodies of interest were anti-*Lp *(SG 1 to 10) and other *Legionella *species (*anti-L.micdadei*, anti-*L.bozemanii*, anti-*L.dumoffii*, anti-*L.gormanii*, anti-*L.jordanis*, anti-*L.longbeachae *SGs 1 and 2, anti-*L.anisa*). All biological analyses were undertaken by the National Reference Center for *Legionella *in Lyon, France. Biological samples were collected and transported to guarantee their stability. The Nancy University Hospital ethics committee approved the study design and the biological sampling procedures.

To characterize exposure, shower water and aerosol of shower have been sampled. Sampling points were chosen in order to be representative of the exposure of the elderly volunteers. All showers could not be sampled, for practical and financial reasons. Thus, a prior evaluation of the hot water system (design, mode of hot-water production, maintenance of point-of-use, water temperature and treatment) was undertaken in each nursing home in order to assess its criticity in terms of *Legionella *risk and to identify the sampling points (showers) that fitted the best with the volunteers' exposure. Accordingly, each nursing home was split, if appropriate, in several sectors. Study participants were allocated to each sector according to where they usually took their shower. One to eight showers were sampled by nursing home.

At the end of the follow-up, sampling of each shower point was performed twice, two days apart; only the hot water faucet was opened to its maximum flow. For each run, two water samples, collected in sterile bottles, were systematically taken: one on the first stream (300 mL); the other (1 litre) after 7 minutes of water draining (which is the average duration of a shower [21]).

Airborne and water *Legionella *was quantified by whole cell fluorescent in situ hybridisation (FISH) using a mix of three specific probes validated for *Legionella *[LEG705, LEG226 and LEGPNE1] [22,23]. In parallel with the FISH detection, the culturable fraction of *Legionella *was evaluated with the standard method (AFNOR NF T 90–431) [24]. The theoretical detection limits of the FISH technique in the study conditions are respectively 3.10^2^/L of air and 9.10^3^/L of water. Its specificity and sensitivity are 72% and 67% [25].

The bioaerosol sampling began only after the faucet of hot water was closed (i.e. after 7 minutes flushing). Two different biosamplers were used: a MAS-100^® ^(impaction on culture medium: GVPC) and an SKC Impinger (impaction on liquid medium: distilled water). *Legionella *count was done by culture using the MAS-100^® ^sampler (two air volumes were collected: 50 L and 500 L), and by culture and the FISH technique using the Impinger (air volume collected: 195 L).

### Case definition

Table 1 summarizes descriptive data from the literature on PF. The symptoms are not specific; there is not to date an agreed clinical definition of PF. Symptoms that predominate in the PF studies are headache, myalgia and fever and, to a lesser extent, shivers, a consequence of infection. These 4 symptoms will be qualified as "major symptoms". Similarly, the other symptoms showed in table 1 will be considered as "minor symptoms".

Based on these literature data, we chosen the interval of 24 to 72 hours after shower as the duration of the incubation period. Hereafter will be qualified as "evocative symptoms of a PF" these major symptoms, possibly associated to minor symptoms, insofar as they have occurred 24 to 72 hours subsequently to a shower. The duration of the PF episode reported in the literature varies from 1 to 5 days; we decided to spread this duration until a new exposure event (eg a new shower) without exceeding a maximal duration of 8 days. A case of PF will be then defined if all the following criteria are met: (i) it occurred within the three days after a shower and exhibited at least one of the major symptoms, associated or not with minor symptoms ; (ii) this episode lasted at least two days, not exceeding eight days ;(iii) since antibiotics treatment could mask some PF symptoms, we included in this definition all symptoms or association of symptoms lasting at least one day among subjects who had taken antibiotics this very day if at least one of these symptoms was a "major" one ; (iv) *Legionella *had to be detected in water and/or aerosol of the index shower that was selected as representing exposure of the case.

We explored two other definitions of a Pontiac episode, in a view to perform a sensitivity analysis. These alternative definitions focus on fever, viewed as the sole major symptom. In the first one, fever could be associated or not with another symptom (among those listed in table 1); in the second, fever was necessarily associated with at least one other symptom. All other criteria (lag after shower, duration of episode and contamination of water and/or of aerosol) were as described above.

### Data analysis

Epi info version 3.3 and SAS were used for data management and analysis. All subjects were ascribed to a shower ward; those having used more than one shower in the course of the study were ascribed to the shower the most contaminated. The exposure value that was retained among the 6 samples available [4 water samples (two runs with two samples: first flow and after draining) and 2 aerosols samples (MAS-100 and SKC Impiger)] was the greatest *Legionella *count that was found, respectively by culture and by FISH.

For antibody titers, different "positivity" thresholds were tested. *Legionella *infection were considered as present beyond increasing sera titers, irrespective of the species or *Legionella *serogroups.

The association between the incidence of PF and contamination of showers were tested with chi squares on incidence rates, and the association with the prevalence of antibody titers by means of the regular chi square test. Tests are one sided, and p values are computed after the exact Fisher test.

## Results

The study population consists in 560 elderly volunteers from 25 nursing homes of Lorraine. Their age and known risk factors are described in table 2. Women represent more than 2/3 of the study population and are, on average, older than male volunteers (p < 10^-4^). Ages ranged from 46 to 102 years, the median value being 81 years. The population is constituted of 12.1% of ex-smokers and 7.5% of current smokers. Three quarters of subjects had a history of at least one previous medical condition (respiratory disorder, conditions impairing immunity or other), without gender difference.

### Incidence of Pontiac fever

**Figure 1:**
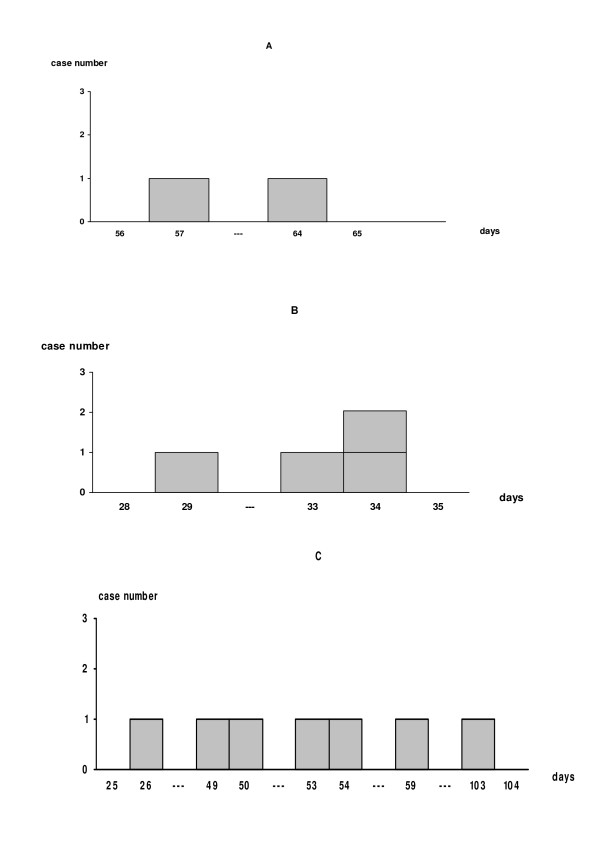
Time distribution of PF cases in the nursing homes were more than one case occurred. A, B and C represent the nursing homes were more than one case occurred. "days" represents the days (since the beginning of follow up) at which Pontiac fever symptoms occurred.

Among the 560 volunteers, 23 subjects had the clinical events corresponding to our definition of PF during the 4 months of follow-up; one subject had twice the clinical event, yielding 24 infectious episodes of PF. The global incidence rate was 0.125 cases per person-year [95% confidence interval: 0.122–0.127]. These cases reside in 13 of the 25 nursing homes. For 10 homes, only one case was reported; two cases occurred in the same nursing home, 4 and 7 cases respectively in two other institutions. In addition to this space clustering, these cases exhibited a time aggregation: less than 10 days separated, respectively, the 2 and the 4 cases that occurred in the same nursing homes. The same held true for 5 out of the 7 cases that occurred in the same nursing home. Figure 1 displays the epidemic curves for these three nursing homes.

The different symptoms presented by cases are displayed in table 3. The incubation period ranged from 24 to 72 hours, with an average and median of 48 hours. Average duration of a PF episode was 4 days. Sixteen subjects (respectively 14) met the two other definitions of a PF episode, yielding incidence rates of 0.08 (0.07) cases per person-year.

### Biological results

Antigenuria was negative for all subjects. The distribution of antibody titers, all *Legionella *species and SGs combined, is given in table 4. Up to antibody titers of 1/128, the distribution of PF cases and of non cases were similar. But subjects with titers of 1/256 had a greater probability to be a case compared to weaker titers (RR = 3.45; p = 0.12); the corresponding antibodies were anti-*Lp *SG 6 and anti-*L.gormanii*. On the other hand, it is noteworthy that 6 subjects who did not meet the definition criteria of PF had titers greater than 1/512 for antibodies anti-*Lp *SG6, anti-*L.jordanis*, anti-*L.micdadei*, anti-*L.dumoffii *and anti-*L.bozemanii*.

### Detection of *Legionella *in shower water

Among the 23 subjects who presented at least one event defined as PF, *Legionella *have been detected in hot water of all the corresponding showers; they have also been found in the aerosol of 7 showers. Aerosolised *Legionella *was always associated with presence of bacteria in the water. Hence, only water contamination will be considered hereafter.

*Lp *was more frequent (66% of positive samples), with mainly *Lp *SGs 2 to 14. According to time of sampling (first flow or after draining) and to the run (first or second run, two days after), *Legionella *counts range from 5.10^3 ^to 2.10^7 ^CFU.L^-1 ^of water (for culture) and from 9.10^3 ^to 15.10^7 ^cells.L^-1 ^of water (for the FISH method). For both detection methods, water *Legionella *concentrations are significantly greater in the first flow than after draining (p = 0.001, paired test).

The association between PF incidence and shower water quality has been studied for different concentration thresholds, according to the analytical techniques that were used (two thresholds for the culture, and three for the FISH technique). Results (table 5) suggest that a contamination level measured by the FISH technique exceeding 10^4 ^*Legionella*.L^-1 ^of water is associated with an increased risk of PF (p = 0.04). The rate of PF is also enhanced for greater contamination levels (RR= 1.83 [not significant] and 2.12 [p = 0.05], respectively for concentrations of 10^5 ^and 10^6 ^*Legionella*.L^-1 ^detected by FISH). On the contrary, no statistically relevant association was observed between the levels of culturable *Legionella *and the PF incidence. This finding suggests a better sensitivity of the FISH technique to characterize exposure to infectious *Legionella*. When the two other definitions of a PF episode are accommodated, levels of 10^4 ^*Legionella*.L^-1 ^of water assayed by the the FISH method are also associated with an increased incidence, although more weakly, with p values of 0.06 (fever associated or not with another symptom) and 0.08 (fever and another symptom) respectively.

### Risk factors

Age, gender or medical history were not associated with incidence of PF, as defined in our study. The only factor that showed a statistically significant association with the "case" condition was immunosuppressive therapy (RR = 4.7, p = 0.02).

## Discussion

This work proposes an operational definition of PF for surveillance and epidemiological investigation. The originality of this study is to lean not on an epidemic situation but on follow-up, during 4 months, of an elderly population residing in nursing homes. Taking a shower was the exposing activity. *Legionella *concentrations were measured both in hot-water and aerosols of showers, using culture and fluorescent in situ hybridisation (FISH) methods. The proposed definition of PF corresponds to the occurrence of at least one of the following "major" symptoms: headache, myalgia, fever or shivers, associated or not with other symptoms labelled as "minor". Such occurrence had to occur 24 to 48 hours after taking a shower contaminated with *Legionella*. The PF incidence is statistically increased when *Legionella *concentrations reach at least 10^4^*Legionella*.L^-1 ^in the shower water, using the FISH method, a level that seems to represent a threshold of risk. We are aware of no published epidemiological study on Legionnaires' disease that evaluated a minimal infective dose. However, a review of Legionnaire's episodes showed that whenever the probable source of contamination was found, water contained more than 10^5 ^CFU.L^-1 ^[2]. Two other definitions of a Pontiac episode were explored that focussed on fever, associated or not with other symptoms; this sensitivity analysis did not alter the results.

The description of PF in our study is unusual in several aspects: the study population and location, the source of the exposure and how it was ascertained.

### Study population and epidemiologic particularities of the results

Our study describes for the first time, to our knowledge, the incidence of PF in a population of elderly people in nursing homes. Previous studies have described epidemic Pontiac episodes with spa (with jacuzzi), decorative fountains [2,5,6,13,26-28], in relation with cooling towers [4], and also among workers involved in cleaning activities with high pressure water in confined spaces [3,17,20,29]. In most studies, subjects (usually young and not living continuously in the setting where exposure took place) were exposed during a short period (generally not exceeding ten days) to a common source, a situation that facilitates linkage between a cluster of symptoms among several individuals and a common source of exposure. On the contrary, our study describes symptoms of PF among subjects who may be exposed iteratively and individually. In this "endemic" context, however, cases appeared on a grouped mode since, besides 10 sporadic cases, all others among 23 who met the case definition occurred on a short period among subjects belonging to three nursing homes. In reference to epidemiologic data available in the literature, the incidence rate calculated in this study expresses this particular context. Indeed, previous papers have described very high attack rates of PF, about 70 to 90% [1] in an epidemic setting. On the contrary, when PF episodes occur in the community in a non epidemic setting, the mild severity of the symptoms can lead to underestimation of true incidence, these banal symptoms being easily overlooked [5]; hence, in a population follow-up setting like in our study, data collection might well miss some cases.

Sera titers of 1/256 appear here as being linked to cases of PF with a relative risk of 3.5 (p = 0.12). Interestingly, this corresponds to the definition of the French National Institute of Health Surveillance (InVS) of a probable case of Legionnaires'disease [30]. Our results concerning the antigenuria (0 positive antigenuria for 23 subjects are similar to those described in some studies about legionellosis [3,10,20]. Six subjects who did not meet the proposed definition criteria of PF exhibited high titers of antibody (> 1/128), an intriguing observation. First, one should consider the small number of cases, allowing for sampling variability. This might also be explained by cross-reactions of antibodies detection in relation with the indirect immunofluorescence technique that is used. These cross-reactions are many and were described for mycobacteries, leptospires, *Chlamydia*, *Mycoplasma*, *Citrobacter*, *Campylobacter *and *Coxiella burnetii *[31]. Moreover, *Legionella *encountered in human pathology generally belong to the *pneumophila *species. In our study, except Lp SG6, the other species observed among subjects with high levels of antibody who did not meet our definition of PF, belong to the non *pneumophila *species (anti-*L. jordanis*, anti-*L. micdadei*, anti-*L. dumoffii *and anti-*L. Bozemanii*). This also can be explained by the possibility of cross-reactions between different serogroups and different species of *Legionella *[32]. The most frequent symptoms that were exhibited by these subjects were cough (5/6), dyspnea (3/6), diarrhoea (3/6) and headaches (3/6). Although symptoms occurred in the three days following a shower, these subjects did not comply with our definition either because they only presented "minor symptoms", or because *Legionella *had not been detected in the showers they used.

In accord with other authors, age, gender and smoking habits are not found as risk factors for Pontiac fever [10,11]. This condition might even affect preferentially young subjects, with typical ages among cases in published epidemics ranging between 30 to 40 years [3,4,10-13]. These figures are not comparable to ours since the average age of our elderly population among cases of PF was 82 years, similar to subjects who did not exhibit PF symptoms.

Among clinical risk factors that have been evaluated in the present study (a history of respiratory conditions, sicknesses impairing the immunity system such as diabetes, cancer, cardiovascular conditions and dementia such as Alzheimer's disease), only immunosuppressive therapy was associated with the incidence of PF (p = 0.02). This very risk factor had been also described in a study where prevalence of community respiratory conditions was compared between subjects residing in nursing homes and at home [33].

### Source and measure of exposure

Aerosolised *Legionella *reached greater concentrations during the first minute, as previously described by several authors [34,35]. One limitation of this study resides in the semi-ecological character of exposure assessment. Indeed, exposure was not assessed at an individual level but by groups of subjects. All showers could not be systematically sampled and we assigned to a shower A (not sampled) the same quality as shower B (sampled) on criteria based on the existence of a common water column feeding the two showers. The resulting exposure misclassification is a Berkson type error [36,37]. It tends to lessen statistical power by increasing the variances estimates of the association; however it does not biase the point estimate, when the average group exposure is correctly represented by the measured concentrations.

Moreover, we have made the hypothesis that the hot-water *Legionella *contamination remained stable during the whole follow-up period. Now, only two measurements of water contamination were done at the end follow-up period in each nursing home to characterize exposure along the study. The validity of this assumption depends therefore on the time variability of the shower water contamination, whose avaluation is under way. It also depends upon other factors such that sensitivity and specificity of the sampling and analytical methods that were used [8,25]. Because the culture method has not the capacity to detect all viable bacteria that are sampled in the environment, due to their very diverse physiological states [38] and to environmental stress, conditions that led to the concept of "viable but not culturable bacteria" [39-41], there is some degree of underestimation of bacterial concentrations. Molecular approaches like the FISH technique might provide more representative concentration estimates, leading to improved exposure assessment to bacterial pathogens. Figures with the FISH method do not exist to date.

## Conclusion

Our epidemiological findings back the operational definition of the PF that we propose. It needs to be confirmed by others studies. The investigation is still going on in order to include a thousand of volunteers, a number that should ensure a greater statistical power.

## Abbreviations

PF : Pontiac fever

Lp: Legionella pneumophila

L. feeleii: Legionella feeleii

L. micdadei : micdadei

L. anisa: Legionella anisa

SG (s): serogroup (s)

anti-Lp: anti Legionella pneumophila

anti-L.: anti Legionella

AFNOR: Agence Française de Normalisation

FISH: Fluorescent In Situ Hybridisation

RR: Relative Risk

CDC: Center of Diseases Control

INVS: Institut National de Veille Sanitaire (National institute of Medical Surveillance)

MAS: Microbial Air Monitoring System

UFC: Colony-Forming Units

* Asterisked abbreviations are not defined in the body of the text

## Financial and non-financial competing interests

The author(s) declare that they have no competing interests.

## Authors' contributions

TP was involved in the epidemiological survey. He performed data statistical analysis and wrote the manuscript.

MAD carried out the environmental analyses (aerosols and water); she participated in its design and coordination and helped to draft the manuscript.

DZN and HP designed and carried out the survey studies, participated in the data analysis and helped for drafting the manuscript.

LM conceived the study, and participated in its design and coordination and helped to draft the manuscript and revising it critically.

All authors read and approved the final manuscript.

## Pre-publication history

The pre-publication history for this paper can be accessed here:



## Figures and Tables

**Table 1:** Clinical data during Pontiac fever episodes described in the literature [references]

Reference	Source of exposure	Incubation period (hours)	Episode (duration)	Symptoms	Type and antibody titers	Legionella species
[2]	Spa with jacuzzi/pool	From 9 to 67 (mean = 38)		**Myalgia**^a^, **Headache**^ba^, Asthenia, **Shivers**, **Fever**, Cough, Nausea, Sore throat^c ^Dyspnea^c^		*L. micdadei*
[3]	Washing with high pressure water			**Fever**^a^, **Shivers**^a^, Asthenia^a^, Thoracic pain, **Headache**^b^**, Myalgia**^b^, Nausea, Cough, Dyspnea, Vomiting, Diarrhoea, Abdominal pain, Ocular redness with photophobia Sore throat	1/128 and 1/256	L. *pneumophila *SGs 1, 3, 4 and 6 and L. *londiniensis*
[4]	Cooling towers	36 to 90	2 - 5 days	**Fever**, Lombago, Arthralgia, **Headache**, Dyspnea, Asthenia Sore throat	Anti-Lp SG71/16 to 1/256	*L. pneumophila *SG7
[5]	Decorative fountains	49	71 hours	**Fever**^a ^**Myalgia**^b^, **Headache**^b^, **Shivers**^b^, asthenia, Back pain Nausea, Cough, Vertigo Cramps Diarrhoea^c ^Vomiting	Anti-Anisa: 1/128 to 1/512	L. *Anisa*
[6]	Spa with jacuzzi/pool		1 - 5 days	Diarrhoea, Vomiting, **Headache**, Nausea, **Myalgia**, Cough^c^	Anti-Lp SG Titer >1/64	*L. pneumophila *SG6
[10]	Cooling towers			**Myalgia**, **Shivers**, Asthenia, **Fever**, **Head ache**	Urinary Ag	L. *pneumophila *SG1
[26]	Spa			**Fever**, **Shivers**, Thoracic pain, Cough, Nausea	Anti-Lp SG6	L. *pneumophila *SG6

a : symptom met in all subjects ; b : symptoms met the most ; c : symptoms met the least;

**Table 2:** Description of the population : age, gender, medical history and smoking status

	Sample size	Mean age (standard-error)	Previous pathologies	Previous immunity conditions	Previous respiratory conditions	Current smokers	Past smokers
Male	158 (28.2%)	77 (± 9)	117 (74.0%)	37 (23.4%)	20 (12.6%)	20 (12.7%)	27 (17.1%)
Female	402 (71.9%)	82 (± 9)	290 (72.1%)	94 (24,3%)	54 (13.4%)	22 (5.5%)	41 (10.2%)
Total	560 (100%)	81 (± 9)	407 (72.7%)	131 (23.4%)	74 (13.2%)	42 (7.5%)	68 (12.1%)

**Table 3:** Frequency of the different symptoms exhibited by the subjects who met Pontiac fever definition.

Symptoms (in bold « major » symptoms	Proportion of subjects (% among 24 episodes)
Vertigo	100%
**Fever**	73%
Cough	54%
Diarrhoea	31%
**Headache**	19%
Asthenia	19%
**Myalgia**	15%
Vomiting	7%
**Shivers**	4%
Nausea	7%
Sore throat	7%
Rhinorrhea	4%
Abdominal pain	4%

**Table 4:** Distribution of antibody titers among cases and non cases of PF, and measure of association.

Antibody titers	Number of subjects with Pontiac fever	Number of subjects without Pontiac fever	RR (and p*)
1/16	5	94	1.39 (0.34)
1/32	2	39	1.27 (0.49)
1/64	3	77	0.94 (0.61)
1/128	2	24	2.08 (0.27)
1/256	2	14	3. 45 (0.12)
1/512	0	6	p = 0.78

* p value are from one-sided tests

**Table 5:** Cases distribution (and person-days at risk) for different levels of shower contaminations and associations' measure.

Contamination threshold	Exposure categories	Number of subjects with Pontiac fever (*person-days at risk*)	RR (and p*)
CFU ≥ 10^3 ^L^-1^	Exposed Unexposed	10 (*24855*) 13 (*42001*)	1.30 (0.33)
CFU ≥ 10^4 ^L^-1^	Exposed Unexposed	9 (*20928*) 14 (*45928*)	1.41 (0.27)
Cells-FISH ≥ 10^4 ^L^-1^	Exposed Unexposed	23 (*56245*) 0 (*10611*)	UD** (0.02)
Cells-FISH ≥ 10^5 ^L^-1^	Exposed Unexposed	19 (*48265*) 4 (*18591*)	1.83 (0.19)
Cells-FISH ≥ 10^6 ^L^-1^	Exposed Unexposed	11 (*20185*) 12 (*46671*)	2.12 (0.05)

*p value are from one-sided tests

**UD: undefined, because of 0 cases among unexposed

CFU: cells count by culture

Cells-FISH: cells count by FISH method
